# Deliberation decreases the likelihood of expressing dominant responses

**DOI:** 10.3758/s13423-020-01795-8

**Published:** 2020-09-11

**Authors:** Torsten Martiny-Huenger, Maik Bieleke, Johannes Doerflinger, Matthew B. Stephensen, Peter M. Gollwitzer

**Affiliations:** 1 Department of Psychology, UiT The Arctic University of Norway, Postboks 6050 Langnes, 9037 Tromsø, Norway; 2 University of Vienna, Vienna, Austria; 3 University of Konstanz, Konstanz, Germany; 4 New York University, New York City, NY USA

**Keywords:** Cognitive control and automaticity, Decision making, High order cognition, Deliberation

## Abstract

Deliberation is commonly assumed to be a central characteristic of humans’ higher cognitive functions, and the responses following deliberation are attributed to mechanisms that are qualitatively different from lower-level associative or affectively driven responses. In contrast to this perspective, the current article’s aim is to draw attention to potential issues with making inferences about mechanisms of deliberation based on characteristics of the observed decision outcomes. We propose that a consequence of deliberation is to simply reduce the likelihood of expressing immediately available (dominant) responses. We illustrate how this consequence of deliberation can provide a parsimonious explanation for a broad range of prior research on decision-making. Furthermore, we discuss how the present perspective on deliberation relates to the question of how the cognitive system implements nondominant responses based on associative learning and affective prioritization rather than voluntary decisions. Beyond the present article’s theoretical focus, for illustrative purposes, we provide some empirical evidence (three studies, *N* = 175) that is in line with our proposal. In sum, our theoretical framework, prior empirical evidence, and the present studies suggest that deliberation reduces the likelihood of expressing dominant responses. Although we do not argue that this is the only consequence or mechanism regarding deliberation, we aim to highlight that it is worthwhile considering this minimal consequence of deliberation as compared with certain higher cognitive functions in the interpretation of deliberation outcomes.

**Electronic supplementary material:**

The online version of this article (10.3758/s13423-020-01795-8) contains supplementary material, which is available to authorized users.

Deliberation—contemplating the pros and cons of a required decision—is an often-observed activity in the process of decision-making and response selection. Furthermore, deliberation is assumed to be a central characteristic of humans’ higher cognitive functions, and deliberation outcomes are often attributed to qualitatively different mechanisms than mere associative thinking. The aim of our present article is to present a different perspective. We postulate that a minimal consequence of deliberation is reducing the likelihood of expressing immediately available, dominant responses. From this perspective, deliberation serves the function of delaying responses and allowing alternative, non-dominant responses to become activated and eventually expressed. We will discuss two major consequences of our perspective: (1) We will highlight a methodological shortcoming in research on deliberation and decision-making: Inferring the mechanisms of deliberation (cognitive processes) on the basis of characteristics of decision outcomes potentially confounds these outcomes with characteristics of the decision task. (2) Within a broader context, we will discuss the consequences arising from the present perspective for the question of whether it is necessary to assume qualitatively different mechanisms for so-called automatic and controlled behavior.

The article is structured as follows. In the *first* part, we present a description of the processes that are responsible for decreasing the likelihood of expressing dominant responses following deliberation. In the *second* part, the empirical part, we demonstrate this minimal consequence of deliberation in an incentivized risk decision-making paradigm. By inducing different degrees of deliberation, we show that more deliberation leads to a decreased likelihood of expressing dominant responses in favor of available alternatives. The *third* part, the discussion, consists of two sections detailing the main conclusions drawn from the present perspective regarding methodological issues when investigating deliberation and apparent qualitative differences between automatic and controlled behavior.

In the following introduction section, we will outline our theoretical perspective based on the following three arguments: (A) Following an event, there is a sequential unfolding of response options with dominant responses being accessible closer in time to the triggering event than others. (B) The activity level of early accessible, dominant responses decays during response delays, giving way for alternative (less dominant) responses to reach the execution threshold. (C) Deliberation induces response delays that provide the minimal conditions (described in A and B) to decrease the likelihood of expressing dominant responses in the respective decision context.

## Sequential unfolding of response options (defining dominant responses)

For a given event, some responses are more likely to reach the execution threshold earlier than other responses. These readily available responses are what we refer to as *dominant responses* (sometimes called prepotent, intuitive, or automatic responses). There is a range of mechanisms that are responsible for making some responses dominant. Most importantly, some responses are more quickly initiated because of an individual’s repeated learning experience of executing a response in a given situation (i.e., associative learning, habits; Wood & Rünger, 2016). Besides this learning-based mechanism, some responses are more quickly expressed because in some way they are compatible with the characteristics of a prior or current simultaneous event (e.g., repetition priming, Logan, 1990; response priming by distractors, Eriksen & Eriksen, 1974; effector–response compatibility, Simon & Small, 1969). Furthermore, responses are dominant because they are in line with certain affective dispositions (e.g., approach or avoidance orientation; Chen & Bargh, 1999). Finally, even in complex problem-solving tasks, probably due to a combination of the previously mentioned mechanisms, the problem at hand can have characteristics that make certain responses more readily available than others (e.g., bat and ball paradigm; Trémolière, De Neys, & Bonnefon, 2014).

Borrowing from the language of diffusion models (e.g., Ratcliff, Smith, Brown, & McKoon, 2016), the listed mechanisms lead to an earlier evidence accumulation start or a quicker evidence accumulation for specific responses. These responses then cross the execution threshold prior to alternative responses with a later starting point or a slower accumulation of evidence. Consequently, the expression of less dominant responses should depend on delays (see Simpson & Riggs, 2007) that allow responses with a later starting point or slower evidence accumulation to reach the execution threshold. In the next section, we will focus on such delays.

## Activation levels of dominant responses decay over time

What happens to immediately accessible, dominant responses if responding quickly is prevented? A series of studies with children (age 3 to 4 years old) deals with this question and indicates that the activation level of dominant responses decays if they are not acted upon. When the children were presented with a task that favored a wrong response, they had trouble not executing this dominant but wrong response (e.g., Simpson & Riggs, 2007). However, in trials in which the children’s opportunity to respond was delayed by a few seconds, they were less likely to execute the dominant (wrong) response, but instead executed the alternative response more often, and thereby committed fewer errors (e.g., Diamond, Kirkham, & Amso, 2002; Simpson & Riggs, 2007; Simpson et al., 2012).

Importantly, Simpson et al. (2012) tested two competing hypotheses of what enables 3-year-old to 4-year-old children to respond more correctly after the induced delay: using the delay for active computation of alternatives (Diamond et al., 2002) versus passive decay of dominant-response activation (Simpson & Riggs, 2007). Simpson and colleagues again used a setup in which the dominant response was the wrong response and induced a short response delay. Importantly, the response delay was filled with a mild distraction task (i.e., children’s thoughts were occupied) or the children were not distracted by an additional task (i.e., children could use the time to “calculate” a response). The results showed that both delay conditions resulted in fewer errors (i.e., fewer dominant responses) as compared with a no-delay condition. This result is surprising from an active computation hypothesis because children who were distracted and could not use the delay for active computation also committed fewer errors. However, it is in line with the idea that inducing a delay results in the decay of a dominant but not executed response (see also Diamond, 2013).

To sum up the argument so far, response options unfold in a sequential order. Inducing a response delay increases the likelihood that early available (dominant) responses start decaying after they have reached their typical activation maximum. Delaying responses provides the chance for the activation level of less-dominant responses to rise beyond that of the dominant response. The evidence summarized so far pertained to induced response delays in laboratory settings. In the next section, we will argue that deliberation may provide these response delays in an everyday context.

## Deliberation delays behavioral responses

In the present research, we define deliberation as the act of focusing attention on the different aspects of a current event and the information activated in memory by that event. Deliberation in the present research is *not* limited to the activation of specific cognitive procedures (i.e., a deliberative mindset; Gollwitzer, Heckhausen, & Steller, 1990) or specific content like analytical reasoning (e.g., Evans, 2006). We conceptualize it as a broad cognitive response that can be initiated like any other behavioral, cognitive, or affective response (see also Doerflinger, Martiny-Huenger, & Gollwitzer, 2017). Consequently, by this definition, deliberation entails delaying any immediate, dominant behavioral responses. We propose that such a delay provides the time for the decay of the activation level of the originally dominant response and at the same time for alternative, less dominant responses to emerge. In other words, whereas deliberation outcomes are typically attributed to specific cognitive mechanisms of deliberation, we propose that the delay alone that is induced by deliberation already has the potential to influence decision outcomes.

In the following section, we will illustrate an issue arising from our perspective for investigating the relation between deliberation and decision-making. The context of decision-making paradigms discussed in the next section allows for a simplification of the presented perspective. The available decision options are typically clearly defined for the decision task at hand. Thus, for the time being, we will omit the question of where alternative, less dominant responses come from (see the Controlled Processes versus Giving Associations the Time to Unfold section for more details on this issue) and focus on typical decision-making paradigms that include response alternatives as the only options from which the research participant can choose.

## Predictions: Decision context and deliberation outcomes

The relevance of our proposal for research on the relation between deliberation and decision-making arises from the predictions that can be made about a systematic relation between the structure of a decision context and the outcome of deliberation. If deliberation systematically decreases the likelihood of expressing dominant responses, predictions about the outcome of deliberation are contingent on the dominant response in each context and the available alternative options. The dominant response aspect depends on the involved psychological mechanisms listed in the first section of this article (e.g., associative learning). The alternative option is especially relevant in scientific investigations because alternative options are defined by the researcher, often in the form of a single alternative.

Imagine a study in which participants are presented with two options (A and B), and Option A is the more dominant response in the given context. According to our present reasoning, the induction of deliberation should decrease the likelihood of choosing Option A. Per the design of the study (Option B is the only alternative), deliberation would lead to an increased likelihood of expressing Option B. Thus, whatever aspects characterize Option B—it could be the normatively more rational, more goal-oriented, or the fairer response—it would be more likely to be selected and lead to differences between a deliberation and no-deliberation condition. However, based on our present perspective, it is unjustified to infer characteristics of the induced deliberation from the characteristics of the observed expression of Option B. This is because the increased likelihood of choosing Option B is a consequence of the context (i.e., what is the dominant response and what alternative option did the researcher implement) rather than the inherent features of Option B. In the general discussion, we review relevant prior research and reinterpret its conclusions based on our present perspective. In the next section, however, we will first present novel empirical evidence for the proposed consequences of deliberation.

## Empirical part

Our present theoretical perspective can be applied to a broad range of decisions and behavioral responses, allowing for a wide range of possible empirical tests. Our current empirical studies are intended to exemplify an instance in which deliberation reduces the likelihood of expressing dominant responses. We adopted an established decision-making paradigm from De Martino, Kumaran, Seymour, and Dolan (2006) in which participants choose between risky and safe options that are framed in terms of losses or gains. This paradigm establishes a decision context with specific characteristics, allowing to test whether deliberation reduces the likelihood of expressing dominant responses. *First*, the decision paradigm includes an affectively driven dominant response (avoiding losses; De Martino et al., 2006) and a single, less dominant, alternative response. *Second*, the paradigm allows including expected value as an indicator of normative decision quality. Critically, it is possible to manipulate the decision quality and the dominant-response factor independently from each other. Thus, the effect of deliberation on dominant responses and normative decision quality can be tested without the danger of confounding both factors.

### Decision paradigm

In incentivized decisions, our participants had to choose between a *safe* and a *risky* option. Whereas choosing the safe option had the guaranteed result of receiving a small share of a predefined resource (i.e., points that translated into monetary disbursement), choosing the risky option resulted in a high probability of receiving nothing, but also a low probability of receiving a large share of the resource. Thus, participants repeatedly chose between a sure but small and a large but risky return. This basic task allowed for further manipulations to induce and measure response dominance and expected value.

### Inducing dominant responses (context framing)

To induce dominant responses and to quantify the number of actually executed dominant responses, we used the so-called framing effect. Framing effects (Tversky & Kahneman, 1981) are systematic response biases depending on how a context is presented, even when there is no factual difference. A common framing is to present outcomes of a decision as losses versus gains for the decision maker. The effect of this difference in the framing is that people show a higher tendency to choose risky options whenever decision outcomes are framed as losses rather than gains (reviewed by Steiger & Kühberger, 2018).

There is evidence that this framing effect is affectively driven (De Martino et al., 2006). Apparently, the aversion of losing something drives decisions towards the option that entails at least a chance of not losing—even if the probability of this option occurring is rather low. Thus, we implemented loss-frame and gain-frame decision trials and operationalized the size of the framing effect—the higher proportion of choosing the risky option under a loss frame compared with a gain frame—as the degree of dominant responding. Based on our theoretical framework, we predicted that a higher degree of deliberation results in a smaller framing effect—fewer expressed dominant responses.

### Varying expected value

To include a second, potentially decision-relevant factor that should be treated independently from the dominant responses, we implemented an *expected value* manipulation. If outcomes are dependent on certain probabilities, expected value reflects the most likely average outcome after a considerable amount of trials. Thus, expected value is the probabilistic value of a given choice. We manipulated expected value on a trial-by-trial basis; for some trials, the expected value was higher for choosing the safe option; for other trials, the expected value was higher for the risky options, and for other trials, both options had the same expected value. From the perspective that participants aimed at maximizing their monetary outcome (something that was reinforced by the task instructions), trial-by-trial choice of the option with the higher expected value can be interpreted as a normatively good choice. We operationalized the expected-value effect—the higher proportion of choosing the risk response in trials with a higher expected value for the risky option compared with trials with a higher expected value for the safe option—as an indicator of decision quality from a normative outcome-maximizing perspective.

Critical for the present theoretical perspective, the secondary factor of expected value was independent from the framing factor (response-dominance manipulation). That is, the dominant response in each trial was equally often of high, low, or equal expected value. From perspectives that relate deliberation or reflective processing to more normative, rule-based, and consequential decision-making (as compared with biased, associative, and experiential; reviewed by Evans & Stanovich, 2013), it could be predicted that the induction of deliberation (in our case “thinking carefully”) increased participants’ consideration of the normative, rule-based, and consequential information (i.e., expected value). Thus, a higher degree of deliberation should result in a higher frequency of responses that are aligned to their expected value. In contrast, our present theoretical perspective merely predicts that deliberation decreases the likelihood of expressing dominant responses. As the expected-value factor does not provide an obvious dominant response, our theoretical framework does not make specific predictions about the consequences of deliberation on the expected value of the responses taken.

The hypothesis that the framing but not the expected-value manipulation affects response dominance is based on the definition of dominant responses, as previously provided in the Sequential Unfolding of Response Options section: Responses driven by the framing effect are based on processing easily detectable cues (e.g., “You will lose . . . .”) and their potential affective consequences (e.g., De Martino et al., 2006). Responses driven by expected value instead require the valuation of outcomes based on calculations that integrate numeric information about absolute values and probabilities. We expect the easily detectable cues of the framing manipulation and the affective responses triggered by the framing to be more quickly accessible than the information provided by calculating the expected value. As the dominant-response factor is a continuum, expected value may provide a dominant response in the absence of other comparatively more dominant decision-relevant factors. In our present studies, however, we assume that response dominance is induced by the more easily comprehensible framing information (e.g., “You will lose . . . .”). Therefore, the reduced likelihood of expressing dominant responses following deliberation should emerge more strongly for the framing than for the expected-value factor.

### The induction of deliberation and framing effects

Previous studies have investigated the amount of information processing (e.g., more or less deliberation) in relation to the framing effect—with conflicting results. Whereas some studies found smaller framing effects following more processing (e.g., Takemura, 1994), other studies found larger framing effects (e.g., Igou & Bless, 2007). The procedures used to manipulate the amount of processing varied considerably between these studies, including the manipulation of motivation, requesting justification, or imposing lengthy time delays (>30 seconds; Igou & Bless, 2007). The inconsistent results are not easily resolved given the diverse deliberation-induction methods. Each method induces additional aspects that go beyond our present conceptualization of deliberation (e.g., justification could induce post hoc reasoning instead of the intended deliberation in advance of a decision). Consequently, we approached the induction of deliberation with a planning procedure that has previously proven to be effective (Doerflinger et al., 2017); it has the advantage of keeping motivation and the actual decision task consistent between low and high deliberation conditions.

In line with research on if–then action planning (i.e., implementation intentions; Gollwitzer, 1999, 2014), participants were asked to adopt an if–then plan in the form of “If I make a decision, then I will think carefully” (inducing deliberation) or “If I make a decision, then I will respond spontaneously” (inducing less deliberation). There is evidence that such if–then planning can be used to vary the depth of thinking (e.g., Bieleke, Gollwitzer, Oettingen, & Fischbacher, 2017; Doerflinger et al., 2017; Wieber, Thürmer, & Gollwitzer, 2015) and amplify information acquisition (Bieleke, Dohmen, & Gollwitzer, 2020). In sum, we present three studies in which we manipulated the degree of deliberation by using a planning procedure. In general, we predicted that a higher induced degree of deliberation results in a smaller framing effect (i.e., leading to fewer dominant responses).

## Studies 1–3: Framing effect and deliberation

### Method

#### Design and participants

All three studies followed a 2 (framing [within]: gain vs. loss) × 3 (expected value [within]: safe vs. equal vs. risky) × 2 or 3 (deliberation [between]; see Table 1 for the specific levels) design. In addition, Studies 2 and 3 included an emphasis factor (within: high vs. low). Participants made binary choices between a safe and a risky option. Proportion of risk responses and decision times were used as the dependent variables. Participants in Study 1 were high-school students in their last or second to last year, and participants in Studies 2 and 3 were university students (see Table 1 for participant-related statistics). We set the number of participants per condition based on prior studies on the effects of deliberation in the context of gain–loss framings. Takemura (1994) and Igou and Bless (2007) performed their analyses on 25–45 participants per condition. Notably, in these prior studies, each participant provided only a single data point (i.e., one response to one decision scenario). In our present research, each participant provided 54 (Study 1) or even 78 (Studies 2 & 3) data points. Because of this significantly larger data basis per participant, we set the targeted sample size to 25 participants per between-participants condition. No analyses on the data of the respective study were conducted before the full reported sample was collected.

**Table 1:** Descriptive participant statistics and design details

	Study 1	Study 2	Study 3
*N* (female)	53 (28)	53 (35)	69 (53)
Age *M* (*SD*)	16.8 (0.8)	22.1 (3.1)	22.1 (5.0)
Age min., max.	16, 19	18, 37	17, 45
Number of decision trials	54	78	78
Deliberation condition levels	Spontaneous vs. Deliberation	Control vs. Deliberation	Spontaneous vs. Control vs. Deliberation

*Note.* From the original 54 participants in Study 1, one participant was removed because the final questionnaire indicated an age below 16 years (local age limit at which using the data would have required parental consent). From the original 54 participants in Study 2, one participant was removed because the experimenter (a research assistant) indicated that the participant appeared to be intoxicated during participation in the study. One demographic questionnaire from Study 1 and three demographic questionnaires from Study 2 are missing due to a failure to retrieve them from a laboratory computer before deletion; these data were coded as missing

#### Procedure

**Fig. 1:**
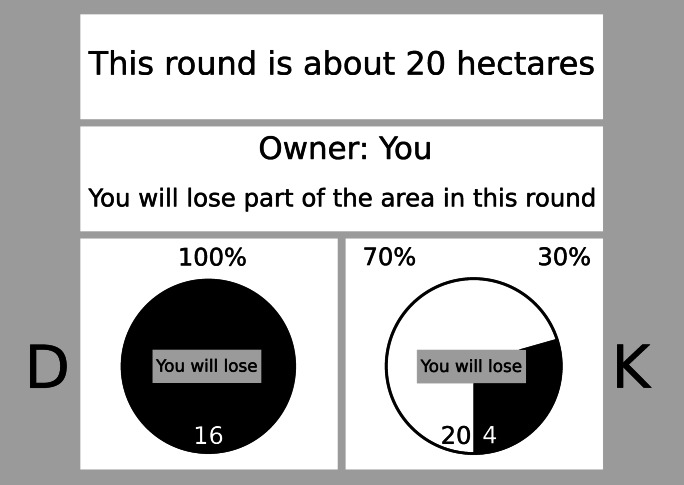
Layout of the information presented in a loss frame decision trial

Participants were informed that the study was about making good decisions in a computerized negotiation about a certain piece of land. The main decision in each trial was deciding between a safe or a risky option. Figure 1 illustrates an example of a loss-framed decision: Choosing the safe option led to receiving a small part (4 hectares, due to losing 16) of the total negotiated piece (endowment; 20 hectares) in the exemplary trial. Choosing the risky option could result in either of two outcomes: With a relatively low probability (30%) participants could receive a larger part (16 hectares, due to losing 4) from the current endowment, otherwise (70%) they received nothing (0 hectares, due to losing 20).

Participants were told that the displayed probabilities reflected the likelihood with which the negotiation opponent would accept or reject the participant’s choice. With regard to the differences between the decision trials, participants were informed that the size of the overall resource (endowment), the probability of the hypothetical negotiation opponent accepting the offer (probabilities of the risk option), and which option (safe or risky) had a higher expected value (i.e., statistically higher outcome likelihood) would vary from trial to trial. Finally, participants were informed that two (Study 1) or four (Studies 2 and 3) decision trials were randomly chosen at the end to calculate their monetary reward for participating in the study (1 hectare of land = 1 point = 0.25 EUR). The last part of the general information was an example illustrating how the task would be presented on the screen, including annotations on the meaning of the different parts of information provided.

#### Deliberation manipulation

Following the general instructions, the induction of the deliberation manipulation started (this part was skipped in the control conditions). Participants were asked to commit to a way of making their decisions by reading and memorizing an if–then response plan. As it is common in if–then planning, the response plans started with specifying a certain situational cue. This cue was different for each Study. The plans started with “If I have to make a decision, . . . .” (Study 1), “If it is a [blue/orange] situation, . . . .” (Study 2), and “If I see an exclamation mark, . . . .” (Study 3). Details about the meaning of these triggers is explained in the Emphasis section below. Importantly, in all three studies, we used the same formulation about the intended response characteristics. To facilitate spontaneous responding, the response plans stated “. . . , then I will respond fast and spontaneously.” To facilitate deliberate responding, the response plan stated “. . . , then I will think carefully.”

Participants were instructed to take some time to memorize the plan. Participants could decide by themselves how much time they spent on doing this, and they pressed a button when they were ready to continue. On the next instruction page, they were instructed to write down the plan on a provided sheet of paper and to call the experimenter who checked the correctness of the plan. In case of an incorrect plan, the experimenter repeated the correct plan before the participant continued with the procedure.

#### Goal-commitment questions (Studies 2 and 3)

Commitment to the goal of making good decisions was relatively high (mean of ~5 on a 7-point scale), and no outliers were detected. Participants answered three goal-commitment questions (plus one question regarding the response plan in the deliberation manipulation conditions). The questions addressed participants’ commitment to making good decisions in the upcoming task on a 7-point Likert scale anchored with 1 (*disagree*) and 7 (*agree*): “I am determined to make good decisions”; “I really do not care about making good decisions” (reverse coded); “It is important to me to make good decisions”; and in the deliberation manipulation conditions, “I am determined to implement the response plan.” Internal consistency of the four commitment questions in the deliberation-manipulation conditions showed a Cronbach’s alpha of 0.65 (Study 2) and 0.79 (Study 3). For the three commitment questions in the control conditions, the Cronbach’s alpha was 0.82 (Study 2) and 0.66 (Study 3). Due to the acceptable internal consistency, a mean commitment score was created for each participant. The mean commitment was relatively high (Study 2: *M* = 5.14, *SD* = 0.47; Study 3: *M* = 5.15, *SD* = 0.35), with no individual outliers detected (Tukey boxplot method).

#### Decision paradigm

Stimulus presentation and response detection was controlled by the open source software PsychoPy (Peirce et al., 2019). Figure 1 depicts an image of the decision task layout. All the information was presented in four white boxes. In the top box, information on the total size of the negotiated resource (endowment) was presented. A second box (the framing box) below the endowment box informed the participants about the owner of the currently negotiated resource. Below this framing box, two squares (decision boxes) placed side by side informed the participants about the to be negotiated share of the resource and the respective probabilities. The letters D and K were displayed to the left and right of the decision boxes to indicate the respective response key. In preparation for the task, participants were asked to place one finger of each corresponding hand on the response keys (D and K, respectively) and start the task whenever they were ready. Each participant completed 54 (Study 1) or 78 (Studies 2 & 3) decision trials. Each decision trial had its own set of properties (combination of endowment, probabilities, framing, etc.). Each participant completed the same set of trials but the presentation order was randomized.

##### Decision framing

One half of the trials was presented in a loss frame, and the other half in a gain frame. Loss frames (gain frames) were established by presenting the information of the respective decision trial in a format that implied that the participant will lose (gain) a part of the resource. This included information that the participant is the owner (is not the owner) of the currently negotiated resource by stating “Owner: You (“Owner: Your opponent”). Furthermore, in the middle of the pie charts illustrating the probability information, it was emphasized that the specified amount of the resource will be lost (gained) when deciding for the respective option by stating “You will lose” (“You will gain”).

##### Expected value

The information presented in the decision boxes varied systematically to establish three conditions differing in which response option had the higher expected value (see Table B in the Supplementary Material). Higher expected value refers to the probability of receiving a higher outcome when choosing a certain response. These probabilities were manipulated to be either in favor of the safe option, the risky option (see Fig. 1; in 10 hypothetical trials, the expected value of always choosing the safe option would result in 40 points, whereas always choosing the risky option would result in 48 points), or neither (i.e., equal; safe and risky option have the same expected value). The higher expected value trials were equally distributed within the total number of trials (Study 1: 18 trials per condition; Studies 2 and 3: 26 trials per condition), with the same amount of loss and gain framings.

##### Emphasis

Studies 2 and 3 included an emphasis that a specific subset of the decision trials was more important than others. The heightened importance of these trials was implemented by making them twice as likely to be chosen for the final reward calculation. In Study 2, participants were informed about these critical trials in written form during the general task instructions. In Study 3, to increase its salience, the information was repeated orally by the experimenter. The critical trials were highlighted by a certain background color (blue or orange, counterbalanced between participants) in Study 2, and by an exclamation mark in Study 3.

The emphasis factor is not directly relevant for our main reasoning. It merely concerns the effectiveness of our deliberation manipulation. The emphasis factor was intended to create a within-participant condition of more or less deliberation in emphasized versus nonemphasized trials. However, the data analysis indicated that the induction of more or less deliberation spilled over to all decision trials (but see the Supplementary Material for a more detailed analysis and some evidence for cue-specific effects on decision times). This is not too surprising considering the repetitive nature of the decision trials and the rather artificial difference between the low and high emphasis trials. As the emphasis factor—even if its implementation would have been successful—does not affect our main conclusions, this factor will only be considered where it is relevant to the description of the methods or statistical analyses.

#### Data preparation

We investigated mean proportions of risk responses and mean response times per participants for each study by creating boxplots. This method revealed no extreme values (i.e., values beyond mean ±3×interquartile range), and thus no participants were removed. Single decision trials were removed if response times were below 500 ms or deviated by more than 3 standard deviations from the mean response time calculated per participant. Due to these criteria, 4.93% of the trials in Study 1, 2.64% from Study 2, and 2.56% from Study 3 were removed.

With regard to the analysis strategy, we investigated participants decisions with a general linear mixed model (*glmer* and *lmer* function of the *lme4* package for R; Bates, Mächler, Bolker, & Walker, 2015) with the choice of the risky option as the dependent variable. We entered expected value, framing, deliberation, emphasis (Studies 2 & 3), their two-way interaction effects and relevant three-way interaction effects as fixed effects and the intercept of participants as random effect. The *p*-value estimates were derived from Wald χ^2^ tests (*Anova* function of the *car* package for R; Fox & Weisberg, 2011) applied to the full model. Confidence intervals for the hypothesis’ critical effects (see Figs. 3 & 4) were calculated from the fitted model (i.e., excluding nonsignificant factors except for those critical for our hypotheses; *confint* function of the *stats* package for R; R Development Core Team, 2019).

### Results (Studies 1–3)

#### Decision response times (deliberation manipulation check)

We consider response times as indicators of more or less deliberation. Thus, participants planning to respond spontaneously should show faster mean response times than participants planning to respond deliberately. The results of the full model analyses of the response times are shown in Table A in the Supplementary Material. At this point, we will focus only on the relevant response time differences for the deliberation condition. Overall, our assumption was confirmed. In Study 1, participants planning to decide spontaneously responded quicker (*M* = 5.55 s, *SD* = 3.83 s) than participants planning to deliberate (*M* = 9.52 s, *SD* = 5.55 s), χ^2^(1) = 11.91, *p* = .001. Similarly, in Study 3, participants planning to decide spontaneously responded quicker (*M* = 6.51 s, *SD* = 2.66 s) than participants planning to deliberate (*M* = 11.02 s, *SD* = 4.79 s), with the control group falling in between (*M* = 9.88 s, *SD* = 5.07 s), χ^2^(2) = 7.47, *p* = .001. Only Study 2 did not provide evidence for a difference between participants in the control group (*M* = 8.46 s, *SD* = 4.30 s) and those planning to deliberate (*M* = 8.46, *SD* = 4.18), χ^2^(1) < 1, *ns*.

There are two features distinguishing Study 2 from Studies 1 and 3. The first concerns the design. In Study 2, we only used a subset (control vs. deliberative) of the design that was used in Studies 1 and 3. The second aspect concerns the response-time data distribution. All three studies show an overall distribution peak of response times around 5 s (see the Supplementary Material for the respective histograms). However, the data of the deliberating participants in Study 2 shows a second, very early peak between 0.5 and 1 s. A similar early peak is only present for the spontaneous participants’ data (as intended), but not for any of the deliberating participants’ data of Studies 1 and 3. To sum up, overall, we have evidence that our manipulations had the intended effect of inducing a low versus high degree of deliberation. However, any specific conclusions drawn from Study 2 must be taken with caution as the response-time data appear to deviate from the overall patterns found in Studies 1 and 3.

#### Decision analysis

The results of the full model analyses are presented in Table 2. There are five consistent observations over all three studies: Three of these observations concern aspects of the decision paradigm (Manipulation Checks section), one observation concerns the predicted Framing × Deliberation interaction (Hypothesis-Critical Effect section), and a last observation concerns the expected-value factor (Factor Unrelated to the Dominant Response section).

**Table 2:** Statistics regarding the full model analysis of the proportion of risk responses

Variable/study		*df*	χ^2^	*p*	*M* (*SD*)	*M* (*SD*)	*M* (*SD*)
Emphasis					Low		High
	Study 1	–	–	–	–		–
	Study 2	1	2.46	.117	0.50 (0.16)		0.47 (0.19)
	Study 3	1	2.93	.087	0.49 (0.16)		0.46 (0.16)
(Higher) expected value					Safe	Equal	Risk
	Study 1	2	93.96	<.001	0.39 (0.15)	0.49 (0.13)	0.60 (0.16)
	Study 2	2	110.89	<.001	0.40 (0.17)	0.48 (0.16)	0.59 (0.18)
	Study 3	2	227.76	<.001	0.36 (0.18)	0.48 (0.18)	0.60 (0.20)
Framing					Gain		Loss
	Study 1	1	80.01	<.001	0.41 (0.16)		0.58 (0.18)
	Study 2	1	41.38	<.001	0.44 (0.18)		0.54 (0.18)
	Study 3	1	85.69	<.001	0.42 (0.18)		0.54 (0.21)
Deliberation					Spon.	Control	Delib.
	Study 1	1	10.99	<.001	0.55 (0.09)	–	0.45 (0.11)
	Study 2	1	0.39	.531	–	0.48 (0.13)	0.50 (0.18)
	Study 3	2	1.17	.556	0.50 (0.18)	0.45 (0.16)	0.49 (0.12)
Emphasis × Expected Value							
	Study 1	–	–	–			
	Study 2	2	0.35	.839			
	Study 3	2	0.55	.761			
Emphasis × Framing							
	Study 1	–	–	–			
	Study 2	1	1.31	.252			
	Study 3	1	1.28	.257			
Emphasis × Deliberation							
	Study 1	–	–	–			
	Study 2	1	0.06	.805			
	Study 3	2	0.04	.982			
Expected Value × Framing							
	Study 1	2	10.90	<.001			
	Study 2	2	13.00	.002			
	Study 3	2	19.55	<.001			
Expected Value × Deliberation							
	Study 1	2	1.26	.532			
	Study 2	2	1.43	.490			
	Study 3	4	2.37	.667			
Framing × Deliberation					(see Fig. 2 for mean proportions)		
	Study 1	1	25.38	<.001			
	Study 2	1	5.04	.025			
	Study 3	2	12.72	.002			

*Note.* Mean proportions and standard deviations were calculated from the means per participant and the respective condition. Spon. = spontaneous; Cont. = control; Delib. = deliberation

#### Manipulation checks

All three studies (see Table 2 for descriptive statistics) showed the expected framing effect, χ^2^(1) > 41.38, all *p*s < .001. Participants were more likely to choose the risky option in loss frame trials than in gain frame trials. Moreover, all three studies showed a main effect of expected value, χ^2^(2) > 93.96, all *p*s < .001. Participants chose the risky option more often if the risky option had a higher expected value than when the safe option had the higher expected value. The proportion of risky responses in the trials with equal expected value fell in between the other two. These two main effects were qualified by a two-way interaction effect between expected value and framing, χ^2^(2) > 10.90, all *p*s < .002. In all three studies, the proportion of responses that were in line with the expected value was greater in gain frame trials than in loss frame trials. In sum, all manipulations affected participants’ behavior in the expected way. The interaction between framing and expected value was unexpected but does not pertain to any conclusions related to how these factors interact with the deliberation manipulation as addressed in the following sections.

#### Deliberation × Framing interaction (hypothesis-critical effect)

**Fig. 2:**
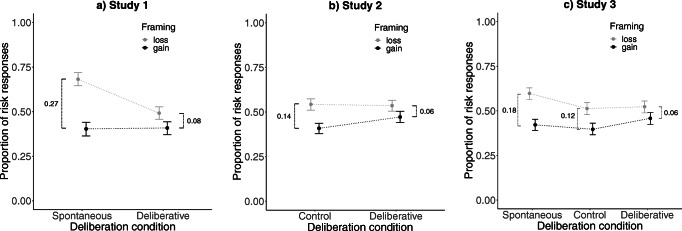
Framing × Deliberation interaction effect for Studies 1 to 3 (whiskers represent the standard error of the mean)

**Fig. 3:**
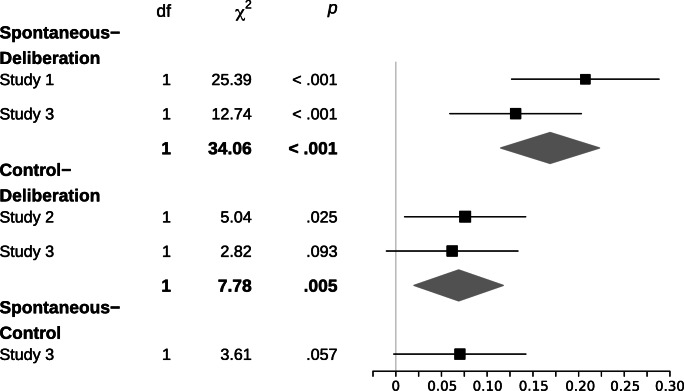
95% confidence intervals for the Framing × Deliberation interaction effect for the five possible tests of comparing the framing effect with the respective low versus high deliberation conditions

We observed a consistent two-way interaction effect between deliberation and framing, χ^2^(1/2) > 5.04, all *p*s < .025. As can be seen in the mean risk response proportions in Fig. 2 and the confidence intervals in Fig. 3, a higher degree of deliberation predicts a smaller framing effect. The observed two-way interaction effects were not qualified by a higher-level three-way interaction effect. In addition, the same Deliberation × Framing interaction effect was found when looking only at the *equal* expected value trials (combined analysis of Studies 1, 2, and 3), χ^2^(1) > 11.34, *p* < .001. This additional analysis reveals evidence for altered responses after deliberation (i.e., less dominant responses) even when deliberation did not allow for the insight that one of the response options was of potentially greater worth. Apparently, the effect of deliberation on framing (i.e., dominant responses) emerged independently of the expected value factor. In sum, given that the framing effect reflects dominant responses, our findings suggest that more deliberation resulted in a decrease in the expression of dominant responses.

#### Expected value (factor unrelated to the dominant response)

**Fig. 4:**
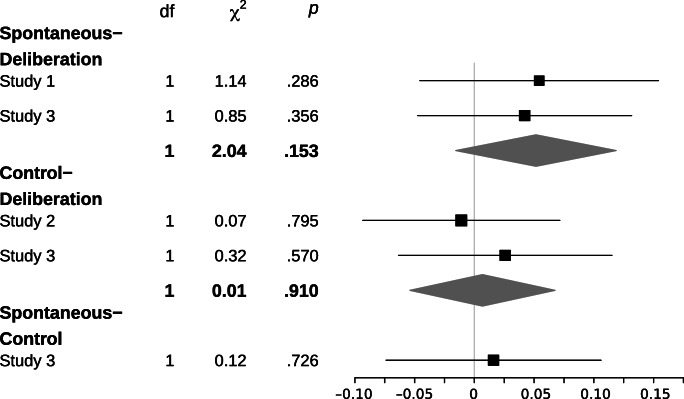
95% confidence intervals for the Expected Value × Deliberation interaction effect for the five possible tests of comparing the expected value manipulation (excluding the equal expected value condition) with the low versus high deliberation conditions

Across all three studies, we found no expected Value × Deliberation interaction effect, χ^2^(2/4) < 2.37, all *p*s > .490. Despite differences in decision times between the different deliberation conditions and evidence that decisions were affected by the deliberation conditions (as reflected in the Framing × Deliberation interaction effect), we found no comparable effects of different degrees of deliberation on considering the expected-value information (the confidence intervals of each possible comparison are displayed in Fig. 4). Even though conclusions drawn from null results can be problematic, the overall pattern of results is in line with our hypothesis that deliberation reduces the likelihood of expressing dominant responses: We found differences in decision outcomes after deliberation for the framing manipulation. We argued that the framing manipulation entails a salient dominant response, and thus the likelihood of showing this dominant response could be reduced. However, we did not find a comparable effect for the expected value manipulation, a manipulation that we argued did not entail a salient dominant response. Thus, with no salient dominant response accessible, the proposed mechanism that reduces the likelihood of expressing dominant responses had no target (we will highlight a similar observation in research using the ultimatum game and the so-called investment game in the Discussion of the Theoretical Relevance section).

## Discussion of empirical findings

Our data from three independent studies provide five consistent observations. *First*, in line with previous research (De Martino et al., 2006), we found the predicted framing effect. In loss frame trials, participants chose the risky option more often than the safe option. *Second*, and most critically, we predicted deliberation would decrease the likelihood of showing the framing effect (i.e., expressing the dominant response). Consistently across all three studies and five individual tests, conditions in which we induced a higher degree of deliberation showed a smaller framing effect (see Figs. 2 & 3). Considering that the framing effect constitutes the dominant-response pattern, conditions of higher degrees of deliberation showed fewer expressions of dominant responses.

*Third*, if the risky option had a comparatively higher expected value, it was more likely to be chosen compared with the safe option; and if the safe option had the higher expected value, the risky option was less likely to be chosen. Thus, across all studies, responses consistently reflected effects of the available information on expected value. This indicates that the expected value information that had to be extracted from a combination of probability and outcome value information was processed in a way that it affected participants’ responses. *Fourth*, the considerable effect of expected value was nonetheless consistently qualified by an interaction with the framing manipulation. In all three studies, the effect of expected value (i.e., higher likelihood of choosing the risky option if it had a higher expected value than the safe option) was smaller for loss frames than for gain frames. There are different ways of interpreting this effect. Most relevant to our present concern, it provides evidence that the implementation of the expected-value manipulation was sensitive to other decision-relevant factors. This is important to consider when evaluating the *fifth* and last consistent observation: We have no evidence (or very little; see Fig. 4 for the confidence intervals of five analyses testing the effect) that the degree of deliberation had an effect on how much the expected value information influenced the decision. From perspectives that relate deliberation or reflective processing to more normative, rule-based, and consequential decision-making (as compared with biased, associative, and experiential; reviewed by Evans & Stanovich, 2013), we could have predicted—and did so before the results of the first study made us consider the present theoretical perspective—that the induction of deliberation (i.e., “thinking carefully”) increased participants’ consideration of the normative, rule-based, and consequential information (i.e., expected value). This, however, was not the case. As drawing a definite conclusion from a nonsignificant result is problematic (e.g., Dienes, 2011, p. 282), it is more worthwhile to highlight the exemplary purpose of varying expected value.

We varied expected value independently from the decision framing that we predicted to determine the dominant response. In the next section, we provide a range of examples where this independence between variables that determine the dominant response and other decision-relevant variables is not given. Thus, conclusions about these other variables are confounded with those that affect dominant responses. In our present studies, we avoided such confounding and detected independent consequences. Deliberation affected the expression of the dominant responses (framing effect), even when analyzing only trials in which the risky and the safe option had *equal* expected values. However, we have no reliable evidence that deliberation affected expected-value considerations—a variable that in comparison with the decision framing is less likely to evoke a dominant response (for a summary of the response time data and a note on the specificity of the planning procedure, see the Supplementary Material).

## Discussion of the theoretical relevance

In the present research, we explored the idea that one consequence of deliberation is reducing the likelihood of expressing dominant responses. Critically, we proposed that this consequence is driven by a mechanism relating to the decision context—what is the dominant response and what alternatives are available—instead of a mechanism related to characteristics of the deliberation process itself. Our discussion section will focus on two implications of this proposal: *first*, its consequences for the (re)interpretation of prior studies investigating characteristics of deliberation, and *second*, for understanding how the cognitive system implements nondominant responses and the distinction between automatic and controlled behavior.

### Context characteristics of the decision task

In the following section, we will provide examples of how our theoretical perspective aligns with previous empirical findings while offering a novel interpretation. A critical aspect of our evaluation of previous studies is that there are always systematic relations between dominant responses and further decision characteristics. These links can have two directions: Normatively low decision quality is linked to the dominant-response option or normatively high decision quality is linked to the dominant-response option.

### The dominant response has low decision quality

We already mentioned decision-making studies with children as participants (e.g., Diamond et al., 2002; Riviére & Lécuyer, 2003). In these studies, the participants were confronted with a context in which habitual responses (i.e., dominant response; saying the word “day” in response to a picture of the sun) were wrong and the alternative, less dominant, response option (saying the word “night”) was correct. The authors observed more correct responses after inducing a response delay. This could merely be a consequence of a mechanism that reduced the likelihood of expressing the dominant response. A similar argument can be made for studies from our own laboratory (Doerflinger et al., 2017). In a poker-like game, we presented participants with a decision to continue with a previously chosen course of action (i.e., dominant response) or to stop. Stopping did not only contradict the participants’ previous decision but also resulted in a sure loss of a previously invested resource. Committing to such a sure loss would result in higher outcomes whenever the likelihood of winning was low (i.e., normatively better decision). Inducing deliberation whenever the likelihood of winning was low (Doerflinger et al., 2017, Study 2) increased the likelihood of stopping. Again, these seemingly better decisions following deliberation can be explained by a mechanism that merely decreases the likelihood of expressing the dominant response (i.e., continued investment)—with the better decision (stopping investment) being the only available alternative to the dominant response of continuing to invest.

The following two examples have a similar setup with dominant responses being the lower quality decision. However, they are noteworthy because our framework can explain additional aspects of the observed results. For example, Neo, Yu, Weber, and Gonzalez (2013) found that inducing a delay (i.e., creating the possibility for more deliberation) changed the participants’ response pattern in the ultimatum game, but not in the so-called investment game. Rejecting unfair offers in the ultimatum game has been linked to affective processes (e.g., Sanfey, 2003). Thus, rejecting unfair offers constitutes the dominant response. In line with our framework, deliberation (induced by a delay) reduced the likelihood of expressing the dominant response (i.e., rejecting unfair offers) in the ultimatum game (Grimm & Mengel, 2011; Neo et al., 2013). Neo et al. (2013) argued that in comparison to the ultimatum game, decisions in the investment game are based on more complex interactions of different motives. From our perspective, this means that the investment game does *not* have a clear dominant response. Thus, in line with our reasoning that the effects of deliberation operate on mechanisms related to the dominant response, inducing deliberation in a context with no clear dominant response will not systematically change the decision outcomes. This—a null effect—was what Neo et al. observed when inducing deliberation in the investment game.

Finally, Obrecht and Chesney (2016) report a study in which inducing deliberation reduced the likelihood of deciding in line with a stereotypical response (i.e., a dominant response; reviewed by Devine & Sharp, 2009) in favor of base-rate information (i.e., probability information on the likelihood of an outcome)—the only available alternative. Importantly, this finding was independent of whether the deliberation instructions pointed participants’ deliberation towards the base-rate information or the stereotypical information. From a saliency perspective, it could be predicted that response options that are in line with the highlighted content of the deliberation process are favored. That is, highlighting base-rate information should increase the use of base-rate information and highlighting stereotypical information should increase the use of stereotypical information for the decision at hand. However, the pattern of results observed by Obrecht and Chesney (2016) is more in line with our theoretical framework: Deliberation (i.e., delay) reduced the likelihood of expressing the dominant response (i.e., the stereotype-based option) in favor of the only available alternative (i.e., the response option that was set up to be in line with the base-rate information).

In sum, these studies qualify as examples in which deliberation (or response delays) produced a range of different—often normatively better—choices. However, these effects of deliberation on decisions are potentially a mere consequence of a mechanism that reduces the likelihood of expressing the dominant response and the fact that the respective task characteristics were set up in a way that the only available alternative was the better choice. Not surprisingly, studies investigating this dominance/low-decision-quality setup are quite frequent because they investigate situations in which people make numerous errors. However, our theoretical framework becomes especially intriguing if it manages to explain results in which the dominance-quality dimension is reversed. We deal with this in the following section.

### The dominant response has high decision quality

One context in which the dominant response is associated with a high decision quality are those in which an immediately available affective response (i.e., dominant response; a feeling of positivity or negativity) towards an object or event provides a good basis for a decision. Wilson et al. (1993) assessed choices to pick a poster that participants would hang up in their living room and measured their satisfaction with it after a few weeks. The choice of a poster that has no other practical value than to be visually pleasing to the owner is a choice that is probably based on the decision maker’s immediate, affective response towards it (i.e., dominant response). Deliberation about the decision should lead to choices that rely less on this immediate affective, dominant response, and therefore lead to a “worse” decision. This is exactly what the results showed with respect to the participants’ satisfaction with the poster after a few weeks. Participants who were instructed to deliberate on the decision were less satisfied with their choice than were participants who were not instructed to deliberate (Wilson et al., 1993). Similar evidence has been presented about preference consistency (Nordgren & Dijksterhuis, 2009) and the topic of motor skills and deliberation (Flegal & Anderson, 2008). Note that this list does not include seemingly related studies on the so-called deliberation-without-attention effect (e.g., Dijksterhuis, Bos, Nordgren, & van Baaren, 2006). Besides doubts regarding the empirical evidence of studies on this topic (see Newell & Rakow, 2011), we believe that typically used tasks to investigate the deliberation-without-attention effect are not informative for evaluating our present proposal, as they are missing a clear dominant response (see also the Related Research section).

Overall, however, if the used decision tasks included clear dominant responses that led to high-quality decisions, inducing deliberation has been shown to reduce the quality of decision outcomes (e.g., Wilson et al., 1993). This outcome is in line with our present perspective, and it highlights the merit of our perspective regarding the much-debated question of whether deliberation is beneficial for decision-making or not (e.g., Dijksterhuis, 2004). From our perspective, the answer is not based on the mechanisms of deliberation, but on the respective context. If the dominant response is the normatively wrong response, then deliberation—resulting in the decreased likelihood of expressing that dominant response—is beneficial. If the dominant response is the normatively good response, then deliberation is more likely to interfere with expressing this good response.

### Limitations of the present framework

Before extending our scope beyond decision-making research, we will highlight some limitations of the present perspective. These limitations pertain to the absence of a dominant response in a decision context, the amount of ambiguity that a decision context is associated with, and the question of where a decision episode ends.

#### The absence of a universal dominant response

If the decision maker does not have a dominant response for the given decision context, the processes described in the present article will predict no effect of deliberation (i.e., no difference in decision outcomes following more or less deliberation). Furthermore, outcomes of experimental studies are usually evaluated based on condition-wise aggregations of a participant sample. For a given context, different individuals can have different dominant responses (e.g., due to different learning experiences). Each individual might follow the pattern that deliberation merely reduces the likelihood of expressing dominant responses. However, the aggregation of the individually different tendencies can result in finding no differences in the aggregated condition effects. Thus, from the perspective of evaluating the outcomes of experimental decision-making studies, our approach allows to make predictions for setups in which dominant responses are a rather universal tendency. Such universal tendencies can have different roots. They can be based on certain motives that are rather fundamental (e.g., wanting to avoid losses) or learning experiences that are shared by most individuals within a given culture (e.g., certain stereotypes).

#### Decision context ambiguity

Our present perspective does not offer systematic predictions whenever various mechanisms (including the one providing the dominant response) favor the same response. Consider for example Study 3 in Doerflinger et al. (2017): Participants planned to deliberate whenever the available information changed (i.e., a new poker card turning up). The likelihood of expressing the dominant response (i.e., continuing with the previously chosen course of action) was not reduced by deliberation if that dominant response was also linked to a high chance of winning (based on the probabilities of the provided numerical information). Basically, all available information—the immediately accessible dominant response to continue to invest and the response provided by a less dominant mechanism of calculating one’s chances of winning—pointed towards the same response of continuing to invest. Thus, the deliberation-induced decay of the immediately available response (i.e., continue to invest) is likely to be prevented by later emerging evidence in favor of the same response. Such situations of various mechanisms favoring a single response could be understood as a situation with low ambiguity. Thus, it appears that to make clear predictions from our present framework, at least some degree of ambiguity is required.

#### Boundaries of a decision episode

The evaluation of our present framework in relation to other studies requires a careful consideration of what constitutes a decision episode. For example, there is research in which participants first indicate an initial decision and then deliberation is induced by giving the participants the chance to reconsider their initial choice (e.g., Shynkaruk & Thompson, 2006). The general tendency to maintain the initial choice (reviewed by Sleesman, Conlon, McNamara, & Miles, 2012) and not switch to an alternative response might be considered as evidence against our present proposal. However, we believe that such a study setup needs to be evaluated as having two decision episodes: making an initial choice and rethinking a previously made choice. The decision options change between these two episodes from “chose A or B” to “chose whether you want to maintain your previous choice or switch to the alternative.” These changed characteristics can result in having different dominant responses in each episode (e.g., Option A is the dominant response in the first episode, whereas maintaining the previous choice is the dominant response in the second episode). Consequently, rather than interpreting the outcome of the request to rethink as being informative of the psychological mechanisms of deliberation, this outcome may again just reflect the expression of another dominant response (i.e., maintaining a previously chosen course of action). The overall numbers of 72% and 88% sticking to their initial choice in Shynkaruk and Thompson (2006) and research on the so-called escalation of commitment phenomenon (Sleesman et al., 2012) seem to be in line with this interpretation. To investigate the mechanisms of deliberation it is not sufficient to compare the responses in the initial, first decision episode to the responses in the second, rethinking episode. Instead, it requires an experimental manipulation of the rethinking episode into a short versus long rethinking episode. Based on our own previous research (Doerflinger et al., 2017), we are confident in predicting that such a setup would result in a pattern that is in line with our present theoretical perspective. In a short rethinking episode, more participants would stick to their previous choice (i.e., expressing the dominant response), and comparably fewer participants would express this dominant response (i.e., more switching) in a long rethinking episode.

In general, our discussion of what constitutes a decision episode highlights that our conceptual framework is not suggesting a “strategy” on how to best make complex, multiattribute decisions (e.g., extensive “elaboration” on what car to buy or which college to attend). Complex decisions are likely to span many decision episodes, and giving such decisions some thought is probably wise (see Newell, 2015). The outcomes of such complex decisions are most likely based on a multitude of different cognitive mechanisms. Instead, our present framework focuses on the psychological mechanisms within the many individual decision episodes that constitute the continuous sequence of everyday behaviors (e.g., habitually grabbing a chocolate muffin in the cafeteria versus deliberating and turning to the less habitual response of grabbing an apple; responding with affectively driven verbal or physical aggression when being provoked versus deliberating and showing a nonaggressive less dominant response). Within each of these episodes, our framework provides predictions and explanations of how nondominant response are expressed.

#### Will the real cognitive mechanism of deliberation please stand up

We want to be clear that we do not claim that our proposed mechanism is the only one that produces decision outcomes following deliberation. We rather assume that it is a series of processes that are most likely active *during* deliberation. The previously described boundaries of the present proposal, however, highlight potential beneficial future directions for deliberation-focused research. Previous and current research on decision-making seems to have a strong focus on situations with clear dominant responses—especially if these dominant responses are normatively of low quality. This focus is reasonable when aiming to better understand the mechanisms that make responses dominant or when trying to help individuals to make better decisions. However, when the interest is in understanding the (higher) cognitive mechanisms of deliberation, we believe that it is not the best possible approach. It involves the potential that observed decision outcomes are erroneously attributed to “higher” level cognitive mechanisms of deliberation instead of the “lower” level processes described in the present research. Using a context that does not include dominant responses should be more informative for understanding deliberation, because it involves less of a risk to confound “lower” and “higher” level mechanisms.

It is noteworthy that there are various studies that resulted in null effect findings when deliberation was manipulated in a context that we would characterize as not including dominant responses (e.g., investment game; Neo et al., 2013; some studies on the deliberation-without-attention effect, Newell & Rakow, 2011; or the expected value manipulation in our present studies). Although there are issues involved in drawing conclusions from null effects, should such null effects appear rather systematically in situations with no clear dominant responses, this would raise questions regarding the assumption that qualitatively different mechanisms are at work in early versus later, more deliberate decisions (see also De Neys & Pennycook, 2019). This also highlights another important limitation of the present article. We do not provide a systematic review of previous studies. Our present focus is on presenting a theoretical framework that, at the very least, provides insights into alternative interpretations of the previously discussed studies. Based on the present theorizing, however, more systematic analyses are warranted that examine decision outcomes following deliberation with a focus on whether the investigated context involved dominant responses or not, and whether conclusions were drawn based on paradigms including either a single or multiple decision episodes.

Another important limitation of the theoretical perspective presented so far will be addressed in the next and final section of this article. So far, we have provided a simplified view that was focused on decision-making research in which alternative response options are often provided with the decision task. In the next section, we will broaden this perspective beyond decision-making research and illustrate how our present conceptual framework is informative regarding the more general question of how the cognitive system implements nondominant responses. To foreshadow our central conclusion, we assume that the same mechanisms that provide the dominant response are also responsible for providing the alternative, less dominant responses if they are given the necessary time (e.g., due to deliberation) to unfold.

## Controlled processes versus giving associations the time to unfold

In this last section, we will return to the question raised at the beginning of the article: Do decisions outcomes following deliberation have to be attributed to qualitatively different mechanisms than the outcome of quickly made decisions? A basic assumption of the so-called cognitive revolution in psychology is that the complexity of human behavior cannot solely be explained by mechanisms we defined here as producing dominant responses like associative learning and affective prioritization (e.g., Miller, 2003). *Control processes* (e.g., Shiffrin & Schneider, 1977) are typically evoked to explain observations that an organism expresses behaviors that go beyond dominant responses—apparently implemented by the organism’s “will” (even if nowadays it is not referred to as “will” anymore; Hommel, 2019). The central proposal of our present research is that merely by inducing a response delay, deliberation systematically influences response outcomes. This is not only relevant in cases where alternative responses are provided by a researcher in a decision-making paradigm but also if response alternatives must be produced by the decision maker. The central idea is that alternatives are not produced by a different system, but instead by the continuation of the mechanisms that also provided the early, dominant responses. We will illustrate this idea based on associative learning as an important mechanism producing dominant responses.

### An example of an associative activation chain

Associative learning mechanisms are often discussed merely at the stimulus (S1)–response (R1) level because R1 is typically executed. However, if R1 is prepared but not executed due to the initiation of deliberation, there is reason to believe that associative spreading of activation continues. In other words, the preparation of R1 can in turn act as a trigger of further concepts; for example, typically perceived consequences of R1. Such associative chains (S1–R1–S2–R2; or simulations) are described in detail in Hesslow (2012). Empirically, there is ample evidence that responses (or their preparation) are not necessarily the end of the associative learning and activation mechanisms. For example, responses can be associatively linked to their observable effects (e.g., Elsner & Hommel, 2001) and a broad range of indirect evidence suggests that response preparation can activate such effect anticipations (reviewed by Hesslow, 2012). These anticipated effects will themselves continue to trigger further associated concepts or responses.

Consider the following example of an individual’s learning experiences. The perception of chocolate muffins (S1) is typically followed by the consumption of a muffin (R1), making R1 a well-learned, habitual response. Assume that in addition to this strong habit, the person makes some novel experiences due to an increased concern about health. From time to time, the consumption of a muffin (R1) is followed by feelings of regret (S2) for having eaten something unhealthy. Such feelings of regret (S2) are usually followed by suppressing the consumption of (more) unhealthy food (R2).

Eventually, if the health concerns remain a chronic goal, the muffins (S1) may at some point become directly linked to “regret” (S2) and consumption suppression (R2; see Moskowitz, Gollwitzer, Wasel, & Schaal, 1999). However, importantly from our present perspective, for a very long time—or forever if the health concerns do not become or remain a chronic goal—consumption (R1) will remain the most dominant response to the muffins (S1). Regret (S2) and consumption suppression (R2) will remain weak associations linked to the consumption response (R1). Consequently, regarding our focus on deliberation, the full chain of associations can only unfold when enough time is at hand. Deliberation time is required in which the consumption response (R1) is prepared—but not executed—and secondary associations can unfold (R1->S2), leading to the consumption suppression response (S2->R2). If this time is not granted, it is likely that the associative chain stops earlier with the expression of the first available response (consumption, R1). Each single step in the chain is the most dominant response with respect to its trigger (S1->R1, R1->S2, S2->R2). Thus, there are no magical leaps that are beyond prior learning experiences. From an observer perspective, however, the observation of the expression of R2 (consumption suppression) in response to S1 (muffins) is the expression of a less dominant response as the obvious learning experience of the individual suggests that R1 (consumption) would have been the most dominant response.

### Choosing the most parsimonious model

Based on this example, do we require assumptions of “cognitive control” to explain the full range of observable behavior? More specific, are there reasons—beyond conventions to label things for communicative purposes—why the associatively activated inhibition of a response at the second level of the activation chain (see also Verbruggen & Logan, 2008) should receive a qualitatively different label (e.g., “control process”) from the equally associatively activated response at the first level of the activation chain (labelled “automatic”)? Beyond this particular example, is it necessary to assume qualitative differences between external (S1) and internal stimuli (preparation of R1 and S2) when they are all processed in the brain—most likely even in the same sensorimotor areas of the brain (Barsalou, 1999)—and all are subject to associative learning?

We approach theory development from the perspective of statistical model development. Assuming qualitative differences in cognitive mechanisms between deliberation and no deliberation, control and automaticity, and internal and external response control significantly increases the amount of assumptions (more parameters making a model less parsimonious). Considerable evidence is required to justify this increase in assumptions. We believe that our present perspective—deliberation inducing delays that allow dominant-response mechanisms to unfold beyond the “first” dominant response—significantly lowers the necessity for assuming a less parsimonious model of the cognitive system.

## Related research

So far, we have not discussed the question of what triggers deliberation. However, our present perspective highlights the importance of this question and the research focused on its investigation. Whether deliberation is triggered or not may prove to be more relevant for understanding response outcomes than the actual mechanisms of deliberation. Regarding our present perspective, it is the question of whether deliberation is triggered or not that determines whether dominant responses are expressed or their likelihood is reduced. Conflict detection mechanisms (e.g., Botvinick, Nystrom, Fissell, Carter, & Cohen, 1999) and the interactions between conflict, affect, and effort mobilization (reviewed by Dignath, Eder, Steinhauser, & Kiesel, 2020) may be the prime candidates for answering the question of when deliberation can be expected to occur.

As laid out in the Introduction, the idea that delays lead to the decay of the activation level of dominant responses has been proposed previously (e.g., Simpson et al., 2012). However, our present proposal extends this assumption in two ways: *First*, we link the idea of delays and dominant-response decay to deliberation. Deliberation acts as the delay-inducing factor in everyday situations that corresponds to the experimentally induced delays in laboratory studies. *Second*, we reevaluate prior studies investigating deliberation in decision-making research and conclude that the delay/dominant-response decay perspective suffices to explain a range of previous empirical observations.

Many of the ideas that are expressed in the present research are influenced by related research, and only in combination can they provide a full view of the perspective that we envision for the cognitive system. In the area of decision-making, De Neys and Pennycook (2019) have highlighted the problems with classical dichotomous thinking of intuition and deliberation. From an action-control perspective, Hommel (2019) has questioned the idea of qualitative differences between automaticity and control. Comparable criticism has been raised about top-down and bottom-up control of visual attention (Awh, Belopolsky, & Theeuwes, 2012). More generally related to our emphasis on associative-learning mechanisms, Abrahamse, Braem, Notebaert, and Verguts (2016) attempted to explain cognitive control by associative learning alone.

Finally, we need to differentiate our approach from proposals that may appear similar. For example, Dijksterhuis et al.’s (2006) unconscious thought theory (or deliberation-without-attention effect) includes a novel mechanism to explain certain deliberation/delay effects on decision-making. Empirically, paradigms typically used to investigate the deliberation-without-attention effect are not necessarily informative to evaluate our present proposal. This is mainly because the investigated context (e.g., processing novel characteristics of a previously unfamiliar product) is usually insufficient to establish a dominant response for one of the choices. Consequently, our present perspective does not predict differences between the typically investigated deliberation/delay conditions. The absence of a strong dominant response is a critical difference to the previously discussed studies that also investigated delays but involved strong dominant responses (e.g., day–night task; Simpson et al., 2012).

Furthermore, there are differences on a theoretical level. Rather than proposing a novel mechanism, we combined previously known mechanisms (e.g., stimulus–response learning, dissipation of the activation level of not executed responses, cognitive simulation) in a novel way. This allowed us to distil a parsimonious mechanism underlying the phenomenon that individuals sometimes appear to express nondominant responses (see also Martiny-Huenger, Martiny, Parks-Stamm, Pfeiffer, & Gollwitzer, 2017, for research with the same aim). Finally, we do not make claims that the processes described in the present research provide better or worse decision outcomes. They provide outcomes that depend on the characteristics of the given situation and prior learning experiences.

## Conclusion

Kahneman (e.g., 2002) compared intuition (i.e., the saliency of a response that is favored by dominant-response mechanisms) with perception. We believe that this is a fitting metaphor. However, we argue that deliberation and deliberation outcomes are comparable with perception as well. The difference in intuition (or automaticity) and deliberation is not in the mechanism, but in “perceiving” something only shortly versus remaining focused on the perception for some time. This perspective led us to propose that a general consequence of deliberation is to decreases the likelihood of expressing dominant responses. Rather passive processes are the basis of this effect. Following an event, deliberation induces a response delay. This delay leads to the decay of the activation of early available, dominant responses while allowing dominant-response mechanisms to continue to produce alternative responses that eventually reach the execution threshold. Empirically, we demonstrated this effect in a paradigm that serves as an exemplary study setup and avoids overlap of dominant responses with other decision-relevant variables. Methodologically, the current research can be considered a cautionary call to not confuse the characteristics of response options favored by deliberation with the mechanisms of deliberation as they can be confounded with the task context. Finally, the described phenomenon allows for understanding behavioral flexibility (expressing nondominant responses) without the notion of voluntary control.

The data for all experiments are available (https://osf.io/wp6eh/?view_only=2d701734309a433b9122a4b4443bc40a). None of the studies was preregistered.

## Electronic supplementary material

ESM 1 (DOCX 101 kb)
