# Supplementary material for: Deliberation decreases the likelihood of expressing dominant responses
Source: Psychon Bull Rev. 2020 Sep 11;28(1):139–57. doi: 10.3758/s13423-020-01795-8 (PMC7870620; doi:10.3758/s13423-020-01795-8)
Supplement: Supplementary file 1 — (DOCX 101 kb) [file 13423_2020_1795_MOESM1_ESM.docx]

# Supplementary Material

## Full Model Analysis of the Response Times (Seconds)

*Table A. Statistics regarding the full model analysis of the decision response times.*

| Variable/Study | | df | F | *p* | *M* (*SD*) | *M* (*SD*) | *M* (*SD*) |
| --- | --- | --- | --- | --- | --- | --- | --- |
| Emphasis | | | | | Low |  | High |
|  | Study 1 | – | – | – | – |  | – |
|  | Study 2 | 1 | 13.24 | < .001 | 8.24 (3.96) |  | 8.95 (4.86) |
|  | Study 3 | 1 | 0.18 | .674 | 8.93 (4.38) |  | 9.39 (5.52) |
| Expected value | | | | | Safe | Equal | Risk |
|  | Study 1 | 2 | 0.97 | .379 | 7.75 (5.36) | 7.80 (5.77) | 7.61 (4.74) |
|  | Study 2 | 2 | 0.02 | .894 | 8.43 (4.26) | 8.47 (4.33) | 8.47 (4.30) |
|  | Study 3 | 2 | 4.24 | .014* | 8.79 (4.60) | 9.48 (4.99) | 8.94 (4.59) |
| Framing | | | | | Gain |  | Loss |
|  | Study 1 | 1 | 37.88 | < .001 | 7.13 (4.68) |  | 8.31 (5.88) |
|  | Study 2 | 1 | 30.59 | < .001 | 8.00 (3.95) |  | 8.94 (4.58) |
|  | Study 3 | 1 | 40.76 | < .001 | 8.55 (4.31) |  | 9.60 (5.18) |
| Deliberation | | | | | Spon. | Cont. | Delib. |
|  | Study 1 | 1 | 11.91 | .001 | 5.55 (3.83) | – | 9.52 (5.55) |
|  | Study 2 | 1 | 0.06 | .809 | – | 8.46 (4.30) | 8.46 (4.18) |
|  | Study 3 | 2 | 7.47 | .001 | 6.51 (2.66) | 9.88 (5.07) | 11.02 (4.79) |
| Emphasis x expected value | | | | |  |  |  |
|  | Study 1 | – | – | – |  |  |  |
|  | Study 2 | 2 | 2.26 | .104 |  |  |  |
|  | Study 3 | 2 | 0.19 | .824 |  |  |  |
| Emphasis x framing | | | | |  |  |  |
|  | Study 1 | – | – | – |  |  |  |
|  | Study 2 | 1 | 1.77 | .183 |  |  |  |
|  | Study 3 | 1 | 2.71 | .100 |  |  |  |
| Emphasis x deliberation | | | | |  |  |  |
|  | Study 1 | – | – | – |  |  |  |
|  | Study 2 | 2 | 0.64 | .423 |  |  |  |
|  | Study 3 | 2 | 22.86 | < .001 | see Figure A in supp. material | | |
| Expected value x framing | | | | |  |  |  |
|  | Study 1 | 2 | 1.83 | .161 |  |  |  |
|  | Study 2 | 2 | 1.25 | .286 |  |  |  |
|  | Study 3 | 2 | 1.02 | .362 |  |  |  |
| Expected value x deliberation | | | | |  |  |  |
|  | Study 1 | 2 | 2.17 | .114 |  |  |  |
|  | Study 2 | 2 | 0.29 | .748 |  |  |  |
|  | Study 3 | 4 | 0.70 | .591 |  |  |  |
| Framing x deliberation | | | | |  | | |
|  | Study 1 | 1 | 0.03 | .869 |  |  |  |
|  | Study 2 | 1 | 1.85 | .174 |  |  |  |
|  | Study 3 | 2 | 4.71 | .009 |  |  |  |
| *Note*. Mean response times and standard deviations were calculated from the means per participant and the respective condition. Spon. = Spontaneous, Cont. = Control, Delib. = Deliberation | | | | | | | |

#

# A Note on the Specificity of the Planning Procedure

Only Studies 2 and 3 allow for conclusions regarding the specific effect of the planning on decision trials that were emphasized by the respective planning cue. Regarding the decision responses, we observed no differences for the emphasized versus normal trials. As commented before, the absence of a significant effect is hard to interpret. Participants completed a large amount of decisions that were very repetitive in their structure, and thus the introduction of an artificial cue to distinguish between emphasized and normal trials may not have been strong enough to lead to any observable differences in the repetitive decisions.

Regarding the decision response times, no emphasis effect or interaction was observed in Study 2. In Study 3, we aimed at making the emphasis factor more salient. Thus, in Study 3, the emphasis factor was not only passively presented within the general instructions, but in addition repeated orally for each participant by the research assistant, including a demonstration of how the emphasized trials will have a larger probability of being selected for the monetary outcome calculation. With this change, in Study 3, we found a pattern beyond the deliberation condition main effect that indicated that the planned responses were additionally facilitated by encountering the respective planning cue. Participants who planned to “think carefully” showed even longer decision response times in trials with the if-then plan cue present than in trials with the cue not present. In contrast, participants who planned to respond “spontaneously” showed even faster response times in trials with the if-then plan cue present than in trials with the cue not present. The latter effect is especially noteworthy as the cued (i.e., emphasized) trials were understood as being more important for the participant’s payoff. Still, participants responded in line with their plan of being spontaneous, indicated by faster response times even on these more relevant trials.

In sum, not finding strong evidence of cue-specific responses in line with the if-then planning may be due to the very repetitive and complex nature of the decision task (complex in comparison to categorization tasks with response times of ~600 ms as compared to the response times in the present studies of ~5-10 s). However, out of the two studies that included a test of cue-specific responses (i.e., inclusion of emphasis factor in Studies 2 & 3), Study 3 that included the strongest manipulation of the emphasis factor, in line with prior studies of if-then planned “modes of thoughts” (e.g., Doerflinger et al., 2017), the response time data showed evidence in favor of the respective response mode being triggered by the critical cue (i.e., if-part of the plan).

##
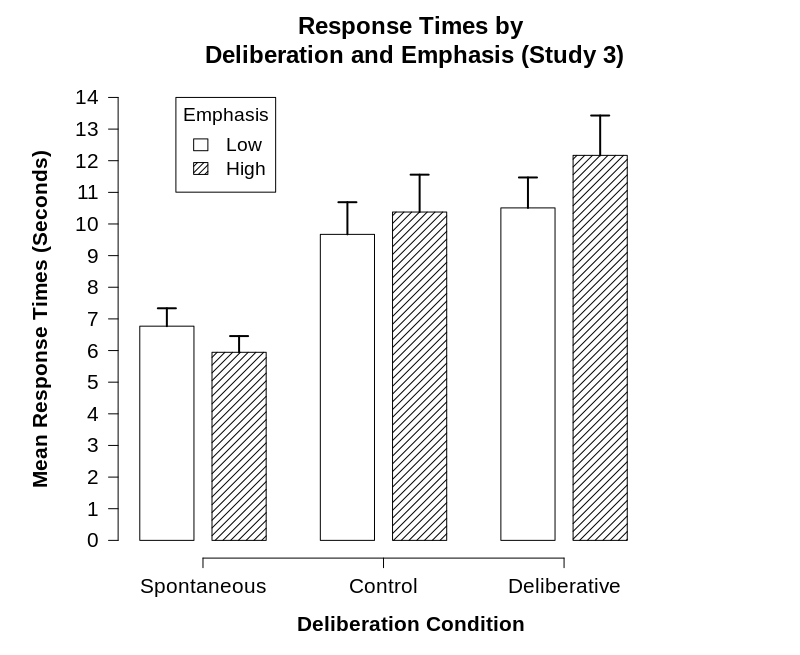


##

## *Figure A.* Deliberation condition by emphasis for decision response times in Study 3 (whiskers represent the standard error of the mean).

##

## *Table B. Ranges of expected value differences for Study 3.*

| Gain Frame | | | | | | | | | | |
| --- | --- | --- | --- | --- | --- | --- | --- | --- | --- | --- |
|  |  | | Gained units if risky outcome is positive | | | Endowment | Proportion of positive risky outcome | | Expected value of risky option | |
|  | Similar expected value | | | | | | | | | |
|  |  | | 40 | | | 50 | 0,1 | | 4 | |
|  |  | | 20 | | | 30 | 0,2 | | 4 | |
|  |  | | 13,5 | | | 20 | 0,3 | | 4,05 | |
|  |  | | 10 | | | 15 | 0,4 | | 4 | |
|  |  | | 8 | | | 12 | 0,5 | | 4 | |
|  |  | | 6,5 | | | 10 | 0,6 | | 3,9 | |
|  |  | | 5,5 | | | 10 | 0,7 | | 3,85 | |
|  |  | | 5 | | | 10 | 0,8 | | 4 | |
|  |  | | 4,5 | | | 10 | 0,9 | | 4,05 | |
|  | Higher expected value of safe option | | | | | | | | | |
|  |  | | 32 | | | 50 | 0,1 | | 3,2 | |
|  |  | | 15 | | | 30 | 0,2 | | 3 | |
|  |  | | 11 | | | 20 | 0,3 | | 3,3 | |
|  |  | | 7 | | | 15 | 0,4 | | 2,8 | |
|  |  | | 6 | | | 12 | 0,5 | | 3 | |
|  |  | | 5,5 | | | 10 | 0,6 | | 3,3 | |
|  |  | | 5 | | | 10 | 0,7 | | 3,5 | |
|  |  | | 4,5 | | | 10 | 0,8 | | 3,6 | |
|  |  | | 4 | | | 10 | 0,9 | | 3,6 | |
|  | Higher expected value of risky option | | | | | | | | | |
|  |  | | 48 | | | 50 | 0,1 | | 4,8 | |
|  |  | | 25 | | | 30 | 0,2 | | 5 | |
|  |  | | 16 | | | 20 | 0,3 | | 4,8 | |
|  |  | | 13 | | | 15 | 0,4 | | 5,2 | |
|  |  | | 10 | | | 12 | 0,5 | | 5 | |
|  |  | | 7,5 | | | 10 | 0,6 | | 4,5 | |
|  |  | | 6 | | | 10 | 0,7 | | 4,2 | |
|  |  | | 5,5 | | | 10 | 0,8 | | 4,4 | |
|  |  | | 5 | | | 10 | 0,9 | | 4,5 | |
| Loss Frame | | | | | | | | | | |
|  | |  | | Lost units if risky outcome is positive | Endowment | | | Proportion of positive risky outcome | | Expected value of risky option |
|  | | Similar expected value | | | | | | | | |
|  | |  | | 10 | 50 | | | 0,1 | | 4 |
|  | |  | | 10 | 30 | | | 0,2 | | 4 |
|  | |  | | 6,5 | 20 | | | 0,3 | | 4,05 |
|  | |  | | 5 | 15 | | | 0,4 | | 4 |
|  | |  | | 4 | 12 | | | 0,5 | | 4 |
|  | |  | | 3,5 | 10 | | | 0,6 | | 3,9 |
|  | |  | | 4,5 | 10 | | | 0,7 | | 3,85 |
|  | |  | | 5 | 10 | | | 0,8 | | 4 |
|  | |  | | 5,5 | 10 | | | 0,9 | | 4,05 |
|  | | Higher expected value of safe option | | | | | | | | |
|  | |  | | 18 | 50 | | | 0,1 | | 3,2 |
|  | |  | | 15 | 30 | | | 0,2 | | 3 |
|  | |  | | 9 | 20 | | | 0,3 | | 3,3 |
|  | |  | | 8 | 15 | | | 0,4 | | 2,8 |
|  | |  | | 6 | 12 | | | 0,5 | | 3 |
|  | |  | | 4,5 | 10 | | | 0,6 | | 3,3 |
|  | |  | | 5 | 10 | | | 0,7 | | 3,5 |
|  | |  | | 5,5 | 10 | | | 0,8 | | 3,6 |
|  | |  | | 6 | 10 | | | 0,9 | | 3,6 |
|  | | Higher expected value of risky option | | | | | | | | |
|  | |  | | 2 | 50 | | | 0,1 | | 4,8 |
|  | |  | | 5 | 30 | | | 0,2 | | 5 |
|  | |  | | 4 | 20 | | | 0,3 | | 4,8 |
|  | |  | | 2 | 15 | | | 0,4 | | 5,2 |
|  | |  | | 2 | 12 | | | 0,5 | | 5 |
|  | |  | | 2,5 | 10 | | | 0,6 | | 4,5 |
|  | |  | | 4 | 10 | | | 0,7 | | 4,2 |
|  | |  | | 4,5 | 10 | | | 0,8 | | 4,4 |
|  | |  | | 5 | 10 | | | 0,9 | | 4,5 |
